# Biobanks in GENETICS and G3: tackling the statistical challenges

**DOI:** 10.1093/g3journal/jkaf060

**Published:** 2025-04-17

**Authors:** Lauren M McIntyre

**Affiliations:** Department of Molecular Genetics and Microbiology, University of Florida Genetics Institute, University of Florida, PO Box 100266, Gainesville, FL 32610, USA

GENETICS is the premier journal for publishing statistical methods. Mapping for quantitative traits was first introduced in GENETICS (Lander and Botstein 1989), as was the concept of a genome-wide significance threshold (Churchill and Doerge 1994). The journal published a model-based clustering method for using multilocus genotype data to infer population structure and assign individuals to populations (Pritchard *et al.* 2000), one of the most insightful approaches to estimating population structure. This was closely followed by a paper describing predictions of phenotype based on high-density molecular marker data (Meuwissen *et al.* 2001).

With the 2011 introduction of G3 into the GSA family, the Society demonstrated its commitment to supporting high-quality, rigorous, and useful science. This has led to G3's publishing of practical solutions for building and genotyping loci de novo from short reads (Catchen *et al*. 2011) and computationally efficient imputation methods in a variety of contexts (Howie *et al.* 2011) as well as R tools for handling extremely large datasets (Grueneberg and de los Campos 2019).

The recent availability of large datasets from biobanks has stimulated a wide array of work on prediction of complex traits in humans, including human height (Lello *et al.* 2018), body mass index (Hoffmann *et al.* 2018), brain images (Wu 2020), coronary artery disease (Zhao *et al*. 2024), and lifespan (Wright *et al*. 2019). These datasets have also given rise to reflections on whether big data can shrink the missing heritability gap (Kim *et al*. 2017), and whether deep learning could improve genomic prediction of complex human traits (Bellot *et al*. 2018). The need for computational efficiency and robust methods in the face of uncertainty continue to be important themes in our pages (Raj *et al.* 2014; Privé *et al*. 2019; Jørsbroe and Albrechtsen 2022; Spence *et al*. 2023). Our ongoing focus on high-quality data and analysis is elegantly illustrated in the January 2025 publication of GenoTools, a Python package that streamlines population genetics research by integrating ancestry estimation, quality control, and genome-wide association studies capabilities into efficient pipelines (Vitale *et al*. 2025).

What can you expect to discover in the April issues of GENETICS and G3? This month, we feature a set of papers that focus on these challenges. Two papers focus on bias: 1) In *“Characterizing selection on complex traits through conditional frequency spectra*,” Patel *et al*. (2024) recognize the biases inherent to GWAS ascertainment, and propose studying the joint distribution of allele frequencies across populations, conditional on the frequencies in the GWAS cohort; and 2) Zhou *et al*., (2025) develop a Bayesian approach to correcting the attenuation bias of regression using polygenic risk score. Their work discusses attenuation bias in the estimation of regression coefficients due to measurement error in polygenic risk score. In “Fast analysis of biobank-size data and meta-analysis using the BGLR R-package leverages sufficient statistics to improve computational speed” (Pérez-Rodríguez *et al*. 2024) the authors' approach enables joint analysis from multiple cohorts without sharing individual genotype-phenotype data and demonstrates how combined analysis can improve the prediction accuracy of polygenic scores. In “SURFBAT: a surrogatefamily based association test building on large imputation reference panels,” the authors address issues of population stratification when individuals are recruited using geographic selection criteria, and they develop an approximation of the TDT that is robust to fine-scale population stratification (Herzig *et al*. 2025). Their approach opens the possibility of efficiently using large imputation reference panels as control groups for association testing.

The recognition that many of the statistical issues in “phenotypic prediction” are shared among organisms (Wray *et al*. 2019) motivates us to continue to encourage methods that improve genomic prediction in crops and animals. Computational efficiency and accuracy of predictions in the presence of genotype-by-environment interaction are computationally challenging. In *Megavariate methods that capture complex genotype-by-environment interactions*, the accuracy and runtime of several different approaches to addressing these issues are benchmarked on simulated scenarios with varying numbers of genotypes and environments (Xavier *et al*. 2024).

Issues in the analysis of large-scale biobank data are important to tackle head on—and in that spirit, we continue to encourage submissions on these topics. As we discuss ongoing challenges and unprecedented opportunities in biobank data, GENETICS and G3 continue to welcome high-quality, thoughtful manuscripts that address these complex issues.
